# An improved 3D-UNet-based brain hippocampus segmentation model based on MR images

**DOI:** 10.1186/s12880-024-01346-w

**Published:** 2024-07-05

**Authors:** Qian Yang, Chengfeng Wang, Kaicheng Pan, Bing Xia, Ruifei Xie, Jiankai Shi

**Affiliations:** 1 Information Technology Center, Taizhou University, 1139 Shifu Dadao, Taizhou City, Zhejiang Province China; 2 College of Mathematics and Computer Science, Zhejiang A & F University, 666 Wusu Street, Hangzhou, 311300 China; 3 Hangzhou Cancer hospital, 34 YanGuan Lane, Hangzhou, 310002 China; 4 School of Computer Science, Hangzhou Dianzi University, Xiasha Higher Education Zone, Hangzhou, Zhejiang 310018 People’s Republic of China

**Keywords:** Brain hippocampus segmentation, MRI, Deep learning, 3D-UNet, Filling technique

## Abstract

Accurate delineation of the hippocampal region via magnetic resonance imaging (MRI) is crucial for the prevention and early diagnosis of neurosystemic diseases. Determining how to accurately and quickly delineate the hippocampus from MRI results has become a serious issue. In this study, a pixel-level semantic segmentation method using 3D-UNet is proposed to realize the automatic segmentation of the brain hippocampus from MRI results. Methods: Two hundred three-dimensional T1-weighted (3D-T1) nongadolinium contrast-enhanced magnetic resonance (MR) images were acquired at Hangzhou Cancer Hospital from June 2020 to December 2022. These samples were divided into two groups, containing 175 and 25 samples. In the first group, 145 cases were used to train the hippocampus segmentation model, and the remaining 30 cases were used to fine-tune the hyperparameters of the model. Images for twenty-five patients in the second group were used as the test set to evaluate the performance of the model. The training set of images was processed via rotation, scaling, grey value augmentation and transformation with a smooth dense deformation field for both image data and ground truth labels. A filling technique was introduced into the segmentation network to establish the hippocampus segmentation model. In addition, the performance of models established with the original network, such as VNet, SegResNet, UNetR and 3D-UNet, was compared with that of models constructed by combining the filling technique with the original segmentation network. Results: The results showed that the performance of the segmentation model improved after the filling technique was introduced. Specifically, when the filling technique was introduced into VNet, SegResNet, 3D-UNet and UNetR, the segmentation performance of the models trained with an input image size of 48 × 48 × 48 improved. Among them, the 3D-UNet-based model with the filling technique achieved the best performance, with a *Dice* score (*Dice* score) of 0.7989 ± 0.0398 and a mean intersection over union (*mIoU*) of 0.6669 ± 0.0540, which were greater than those of the original 3D-UNet-based model. In addition, the oversegmentation ratio (*OSR)*, average surface distance (*ASD*) and Hausdorff distance (*HD*) were 0.0666 ± 0.0351, 0.5733 ± 0.1018 and 5.1235 ± 1.4397, respectively, which were better than those of the other models. In addition, when the size of the input image was set to 48 × 48 × 48, 64 × 64 × 64 and 96 × 96 × 96, the model performance gradually improved, and the *Dice* scores of the proposed model reached 0.7989 ± 0.0398, 0.8371 ± 0.0254 and 0.8674 ± 0.0257, respectively. In addition, the *mIoUs* reached 0.6669 ± 0.0540, 0.7207 ± 0.0370 and 0.7668 ± 0.0392, respectively. Conclusion: The proposed hippocampus segmentation model constructed by introducing the filling technique into a segmentation network performed better than models built solely on the original network and can improve the efficiency of diagnostic analysis.

## Introduction

Brain metastases are becoming an increasingly common complication of systemic cancers [1, 2]. Approximately 20–40% of patients with extracranial tumours develop cerebral metastases during the course of the disease, often leading to a poor prognosis [3, 4]. Currently, radiation therapy, including whole-brain radiation therapy (WBRT) and stereotactic radiosurgery (SRS), is still the primary treatment for both the prevention and treatment of intracranial metastases [5]. Unfortunately, patients with tumours undergoing radiation therapy often suffer brain injuries caused by radiation. Clinical trials by WBRT RTOG 0212 [6] and RTOG 0214 [7] have demonstrated that there is a significant decline in neurocognitive or intellectual function after receiving WBRT. In addition, the greater the dose is, the more severe the decrease. A study by Eric L Chang et al. indicated that patients receiving a combination of SRS and WBRT have a significantly greater risk of cognitive impairment than those who are treated with SRS alone [8]. In recent years, increasing attention has been focused on neural stem cells, which are critical sites susceptible to radiation-induced injuries [9]. Neural stem cells are located mainly in the brain hippocampus; these cells strongly proliferate and migrate and can serve as reserves, participating in the repair of cerebral lesions [9]. The subgranular zone of the hippocampus is a crucial neural centre for learning and memory [10]. Many clinical studies have demonstrated that there is a significant association between the hippocampus and cognitive state [11]. When killing tumour cells, radiation therapy can also inadvertently affect surrounding healthy cells, leading to undesirable side effects [12]. Bilateral or unilateral radiation-induced injury to the hippocampus can influence the processes involved in learning and memory formation [13]. This can lead to cognitive functional disorders and can significantly influence patients’ quality of life [14]. Therefore, accurate delineation of the hippocampus region is crucial for decreasing the radiation dose and minimizing radioactive damage during radiotherapy [15, 16]. However, it is challenging to accurately delineate the hippocampus region in MR images due to the low signal-to-noise ratio (SNR), the poor quality of MR images, and the small size of the hippocampus in MR images [17]. In addition, among all brain tissues, the hippocampus is the most susceptible to ageing as life spans increase. Recent studies have shown that a decrease in hippocampal function is primarily attributed to the death of brain neurons [18, 19]. Moreover, numerous studies have shown that abnormalities in the hippocampus are closely related to the pathogenesis of epilepsy, intellectual impairment, Alzheimer’s disease and other neurological pathologies [20–23]. Therefore, it is important to accurately recognize the hippocampus region for the prevention and early diagnosis of neurosystemic diseases. As a result, developing faster and more precise hippocampus segmentation methods for MR images has become a critical challenge.

Pruessner et al. proposed a manual hippocampus segmentation method, but it has limitations such as low efficiency, low accuracy and a high degree of subjectivity [24]. Strck et al. proposed a semiautomatic hippocampus segmentation method, which improved the efficiency of segmentation compared to manual methods. However, the accuracy of this segmentation method is still limited and cannot meet the demands of clinical application [25]. To achieve both high efficiency and high accuracy for hippocampus segmentation, several automatic segmentation methods based on deformation models [26–28], mapping technology [29–32] and machine learning [33] have been proposed. These methods have made significant advancements in hippocampus segmentation but still face challenges in accurately distinguishing the hippocampus from other tissues with similar grey values in MR images.

With the rapid development and application of deep learning in the field of image processing, many segmentation networks have been developed [34–36]. Ciresan et al. applied convolutional neural networks (CNNs) to segment the neuron cell membrane and achieved ideal segmentation results [37]. CNNs have been used for segmentation tasks in brain tumours [38], retina [34, 39], interstitial and epithelial tissues [35], liver tumours [40], and lung parenchyma [41]. For the segmentation of the hippocampus, Chen et al. used U-Seg-Net to segment the hippocampus with 2D sections of MR images first, and then 3D segmentation results were obtained via reconstruction of 2D segmentation results [42]. Liu and Yan combined deep learning and the lattice Boltzmann model to segment the hippocampus on MR images [43]. A U-Net architecture was proposed by Weng et al. and applied to image segmentation [44].

**Fig. 1:**
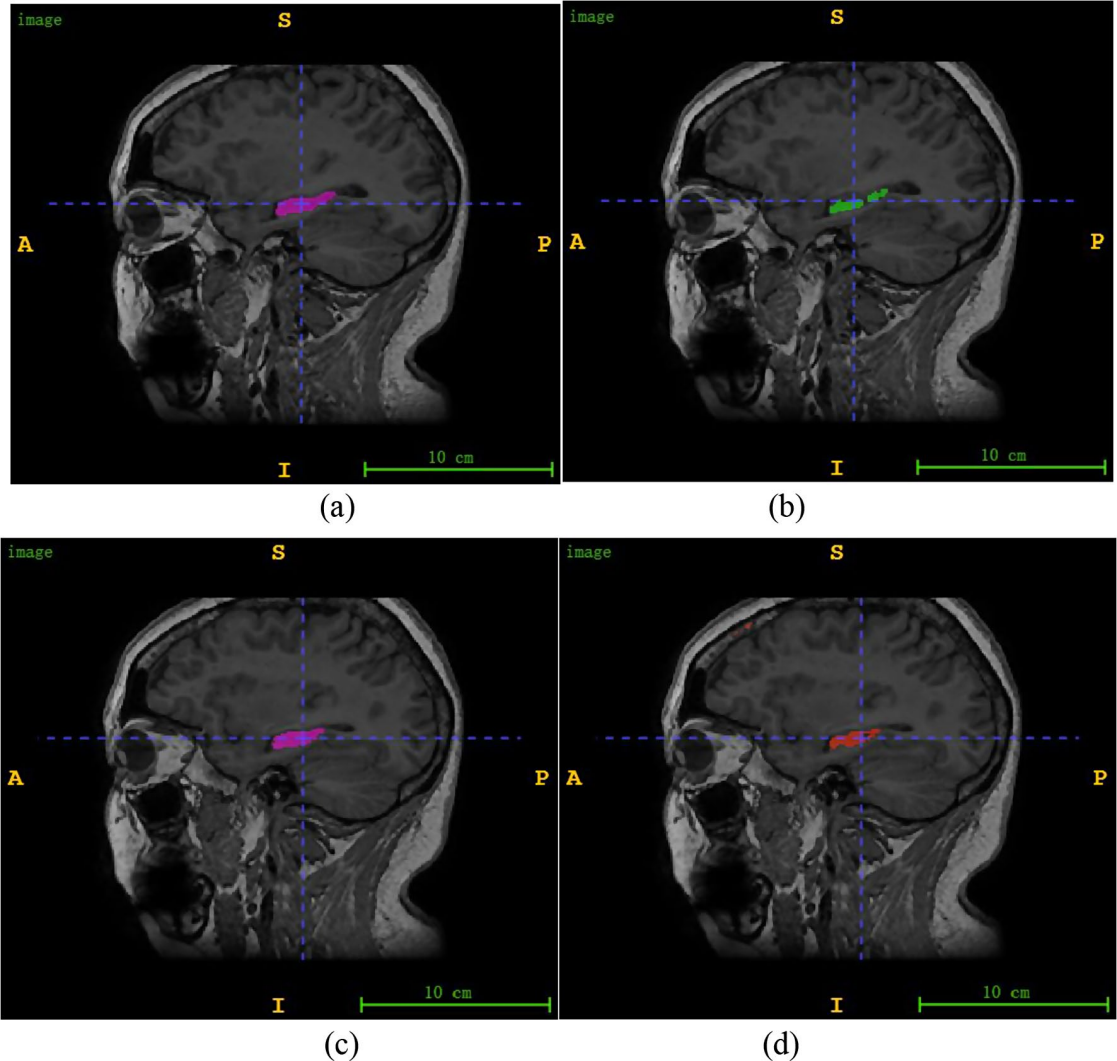
**(a)** and **(c)** Ground truth for two cases; **(b)** and **(d)** Cases of ‘discontinuous segmentation’ and ‘hollow segmentation’, respectively

These region of interest (ROI) segmentation methods achieve high accuracy, but these models were trained with 2D slices of MR images, ignoring the correlations between slices in 3D images, which can yield discontinuous and unsmooth results during segmentation [45]. To overcome this defect, Cieck et al. proposed a 3D-UNet segmentation method to detect the hippocampus with MRI [46]. This method is directly based on voxel-scale information and requires many parameters to be trained. In addition, the distribution of the target region is often discontinuous, leading to discontinuous segmentation or hollow segmentation, as shown in Fig. 1 (b) and (d), respectively.

**Fig. 2:**
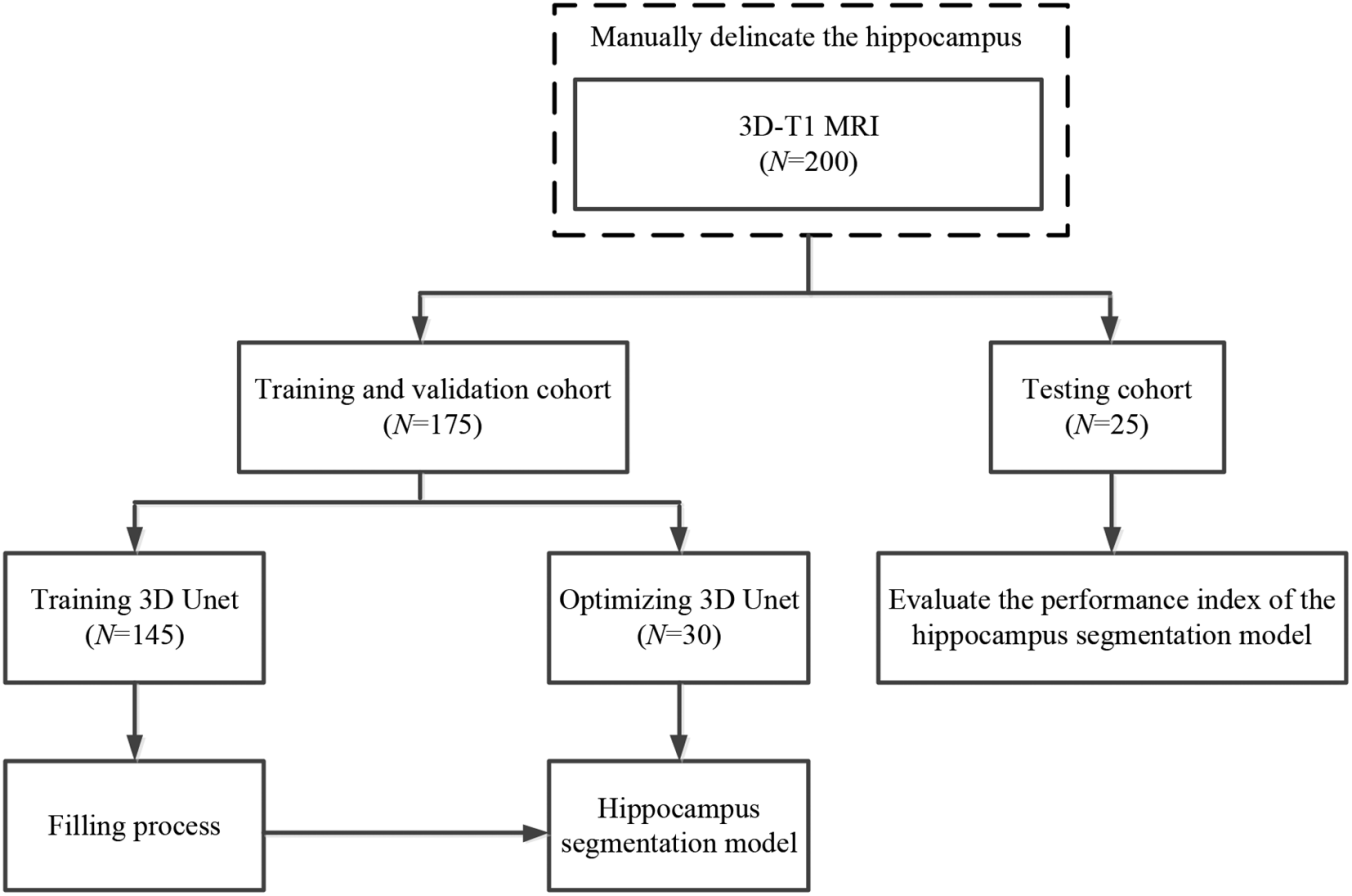
The methodology of this study

Consequently, in this study, we introduce a filling technique into a 3D-UNet model to segment the hippocampus from MR images collected from patients who underwent 3D-T1 magnetic resonance imaging of the brain. The methodology of this study is illustrated in Fig. 2.

## Data and methods

### Datasets

#### Magnetic resonance images

In this study, we collected 200 three-dimensional T1-weighted (3D-T1) sequence MRI scans without gadolinium contrast enhancement. This image dataset was acquired from 200 patients who underwent 3D-T1 sequence MRI scanning from June 2020 to December 2022 at Hangzhou Cancer Hospital. These data were divided into three groups for model training, validation and testing, with images for 145, 30 and 25 patients, respectively. The images for the first group of 145 patients were used to train the hippocampus segmentation model. The images for the second group of 30 cases were used to fine-tune the hyperparameters of the model. The images for the third group of 25 cases were used as the test set to evaluate the performance of the segmentation model. All patients were adults over 18 years of age, and MRI confirmed that the hippocampus was not affected by any disease. The hippocampus was delineated manually from MRI scans by three deputy chief physicians following the RTOG 0933 hippocampal delineation guidelines [47, 48]. The 200 patients were numbered in sequence from 001 to 200. Images for patients who were numbered 001 ∼ 070, 071 ∼ 140 and 141 ∼ 200 were manually segmented by the first, second and third deputy chief physicians, respectively.

### Automatic hippocampus segmentation model

A filling technique was introduced to 3D-UNet to establish an automatic hippocampus segmentation model. The details are as follows.

#### 3D-UNet model

**Fig. 3:**
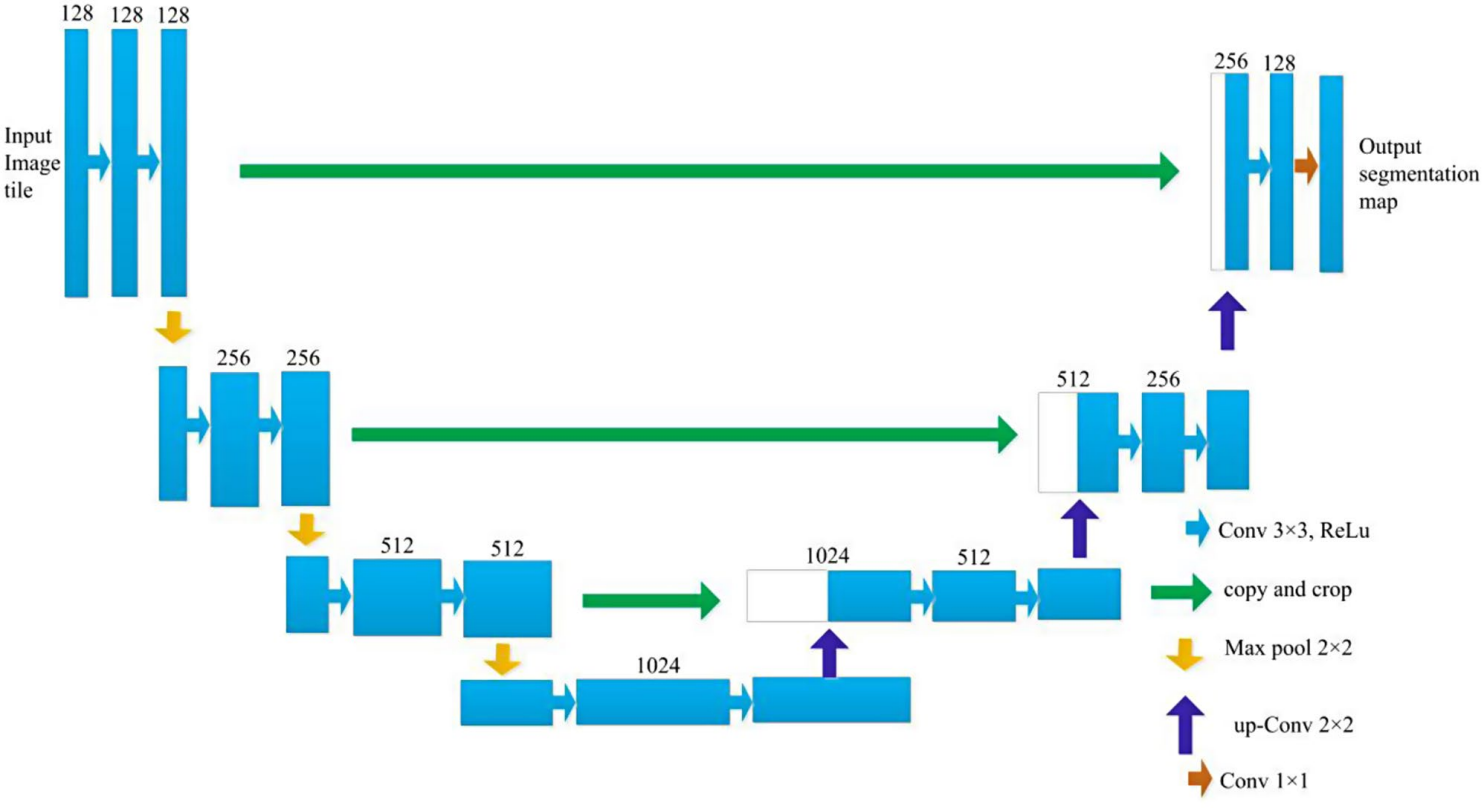
Structure of 3D-UNet

3D-UNet is a deep convolution neural network composed of an analysis path and a synthesis path, and each path has 4 resolution layers. The network structure of the 3D-UNet model is shown in Fig. 3.

The input of the network was a 128*128*128 voxel tile of an image with 3 channels. In the analysis path, each layer consists of two 3 × 3 × 3 convolution layers that are activated by a rectified linear unit (ReLU); then, a 2 × 2 × 2 max pooling operation with a step size of 2 is performed. In the synthesis path, each layer is composed of an upconvolution operation with a kernel size of 2 × 2 × 2 and a step size of 2; then, two 3 × 3 × 3 convolution layers activated by a ReLU function are used. In the analysis path, layers with matching resolutions are connected via a shortcut, which provides the essential features for reconstruction. In the final layer of the synthesis path, the number of output channels is reduced to match the required number of output feature map channels using 1 × 1 × 1 convolution. This architecture design enables highly efficient segmentation with relatively few annotated images by utilizing a weighted soft-max loss function. This approach has demonstrated excellent performance in various biomedical segmentation applications.

#### Filling technique for the segmentation of the hippocampus

In this model, a sliding window with a size of 96 × 96 × 96 was used to identify the hippocampus from 3D-T1 sequence MR images. However, challenges were encountered due to the misrecognition of scattered voxels and the presence of continuous noise points in the image space. As a result, a maximum connected region algorithm was introduced to mitigate the influence of noise points on the recognition results. Additionally, a discontinuous distribution in the brain shell region often leads to the appearance of holes in the recognition outcomes. To address this issue, a filling technique was introduced to improve the performance of the segmentation model. Specifically, in each layer, for any two points *P*_1_ and *P*_2_, with coordinates of (*x*_1_, *y*_1_) and (*x*_2_, *y*_2_), respectively, if the points are within a certain threshold range, i.e., the coordinates of points *P*_1_ and *P*_2_ satisfy formula (1), then a connection path is present.

1 $$\left({x}_{2}-{x}_{1}+{y}_{2}-{y}_{1}+2\right)\le \theta$$

**Fig. 4:**
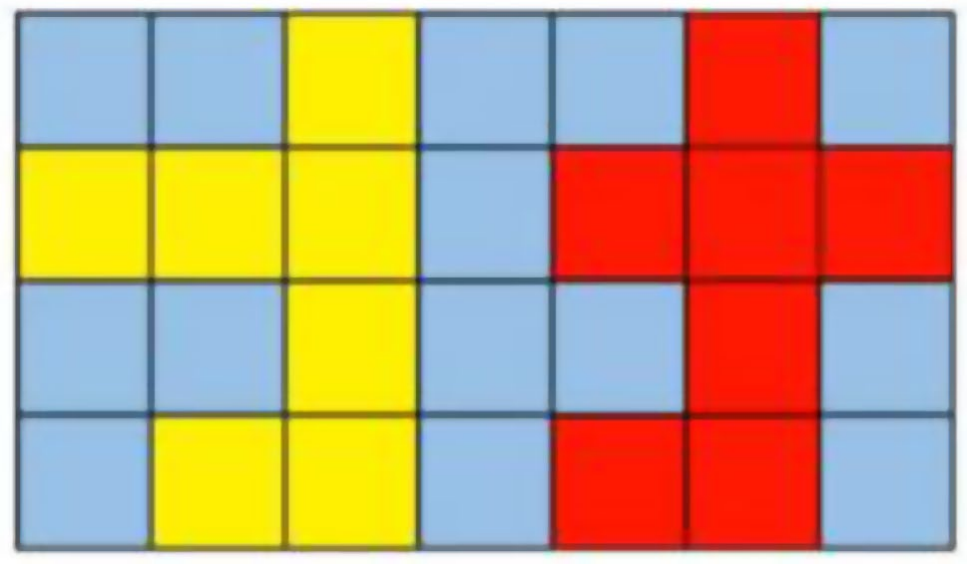
Illustration of the filling technique

*θ* is the threshold value. An illustration of the filling technique is shown in Fig. 4. When *θ* is set to 1, the yellow region and the red region are two independent regions. When *θ* is set to 3, the yellow region and the red region are connected, forming one region, thus eliminating the disconnection between the two regions.

### Training

Image enhancement was performed based on scaling, rotation and grey value augmentation in this study. In addition, a smooth dense deformation field method was used for both ground truth labels and image data. Specifically, random vectors were sampled with an interval of 32 voxels in each direction from a normal distribution. Then, *B*-spline interpolation was used. Due to the small proportion of ROIs in brain MR images and the imbalance among pixel categories between ROIs and background areas, network training with any loss function alone cannot achieve ideal results. As a result, a weighted cross-entropy loss function was used, in which the background weight was frequently decreased and the ROI weight was increased to overcome the imbalance between the pixel area of ROIs and the background region. The intensity of the input data was transformed to a range of [0, 500], which was found to provide the best contrast between the background and ROIs. Data augmentation was carried out in real time, producing a variety of different images for training iterations.

### Evaluation indices for the segmentation model

To evaluate segmentation model performance, the *Dice* score [49–53], intersection over union (*IoU*) [54], oversegmentation ratio (*OSR*) [53], undersegmentation ratio (*USR*) [53], average surface distance (*ASD*) [49] and Hausdorff distance (*HD*) [49] were used in this paper, as shown in formulas (2) to (7), respectively.

The *Dice* score is one of the most commonly used metrics for assessing medical volume segmentation models [49–53]. The definition of the *Dice* score is shown in formula (2).

2 $$Dice\left(GT,Pred\right)=\frac{2are{a}_{GT}\cap are{a}_{Pred}}{are{a}_{GT}+are{a}_{Pred}}$$

where ${area}_{GT}$ is the pixel area of the hippocampus in ground truth images, as delineated manually by a deputy chief physician. ${area}_{Pred}$ is the pixel area predicted with the segmentation model.

The intersection over union (*IoU*) is used to measure the accuracy of the segmentation model and quantify the degree of similarity between the annotated ground truth data and the region segmented with the model. The definition of the *IoU* is shown in formula (3).

3 $$IoU=\frac{TP}{TP+FP+FN}$$

In formula (3), *TP* is the pixel area of true positives, *FP* is the pixel area of false positives, and *FN* is the pixel area of false negatives.

The current medical image segmentation approaches have limitations in effectively solving the problems of oversegmentation and undersegmentation [50–53]. The metrics used to evaluate oversegmentation and undersegmentation focus on the proportions of incorrectly segmented and unsegmented pixels, which can reflect the performance of the segmentation model in detail [53]. The oversegmentation ratio (*OSR*) and undersegmentation ratio (*USR*) are defined in formulas (4) and (5), respectively.

4 $$OSR\, = \,\frac{{FP}}{{{R_s}\, + \,{T_s}}}$$

5 $$USR\, = \,\frac{{FN}}{{{R_s}\, + \,{T_s}}}$$

In formulas (4) and (5), *FP* is the pixel area of false positives, and *FN* is the pixel area of false negatives. *R*_*s*_ refers to the reference area of the ground truth ROI, which is delineated manually by a deputy chief physician, and *T*_*s*_ refers to the pixel area of the hippocampus estimated with the segmentation model.

Spatial distance-based metrics such as average surface distance and Hausdorff distance are widely applied to assess the performance of segmentation models. In this study, the average surface distance and Hausdorff distance were used to evaluate the performance of the segmentation model. The definitions of the average surface distance and Hausdorff distance are shown in formulas (6) and (7), respectively.

6 $$\begin{aligned} ASD\,\left( {A,\,B} \right)\, & = \,\frac{1}{{S\left( A \right)\, + \,S\left( B \right)}}\, \\ & \left( {\sum\limits_{{}_{{}^SA} \in S\left( A \right)} d \,\left( {{s_A},\,S\left( B \right)} \right)\, + \,\sum\limits_{{}_{{}^SB} \in S\left( B \right)} {d\left( {{s_B},\,S\left( A \right)} \right)} } \right) \\ \end{aligned}$$

In formula (6), *S*(*A*) and *S*(*B*) are the sets of surface voxels of *A* and *B*, respectively. *d* (*s*_*A*_, *S*(*B*)) indicates the shortest distance from an arbitrary voxel *s*_*A*_ to *S*(*B*). *d* (*s*_*B*_, *S*(*A*)) indicates the shortest distance from an arbitrary voxel *s*_*B*_ to *S*(*A*).

7 $$H\,\left( {A,\,B} \right)\, = \,\max \,\left( {h\left( {A,\,B} \right),\,h\,\left( {B,\,A} \right)} \right)$$

where *h*(*A*,*B*) and *h*(*B*,*A*) are the one-way Hausdorff distances between (*A*, *B*) and (*B*, *A*), respectively, as shown in Eqs. (8) and (9).

8 $$h\left( {A,\,B} \right)\, = \,\mathop {\max \,}\limits_{a\, \in \,A} \,\left. {\left( {\mathop {\min }\limits_{b\, \in \,B} } \right.\,\left\| {a\, - \,b} \right\|} \right\}$$

9 $$h\left( {B,\,A} \right)\, = \,\mathop {\max \,}\limits_{b\, \in \,B} \,\left. {\left( {\mathop {\min }\limits_{a\, \in \,A} } \right.\,\left\| {b\, - \,a} \right\|} \right\}$$

## Results and analysis

### Experimental environment

In this study, we carried out the experiments in a Windows system environment. Table 1 shows the information for the hardware environment. Table 2 shows the information regarding the model parameters.

**Table 1:** Experimental environment configuration

Name	Configuration
GPU	NVIDIA 2080 Ti
CPU	I9 13,900 k
MONAI	0.8.1.
Memory	128G
Operating system	Windows 11

**Table 2:** Experimental model parameters

Training parameters	Values
Input image size	48*48*48
Batch size	8
Epochs	600
Optimizer	Adam
Learning rate	0.001

### Comparison of the performance of hippocampus segmentation models established with different deep learning networks

**Fig. 5:**
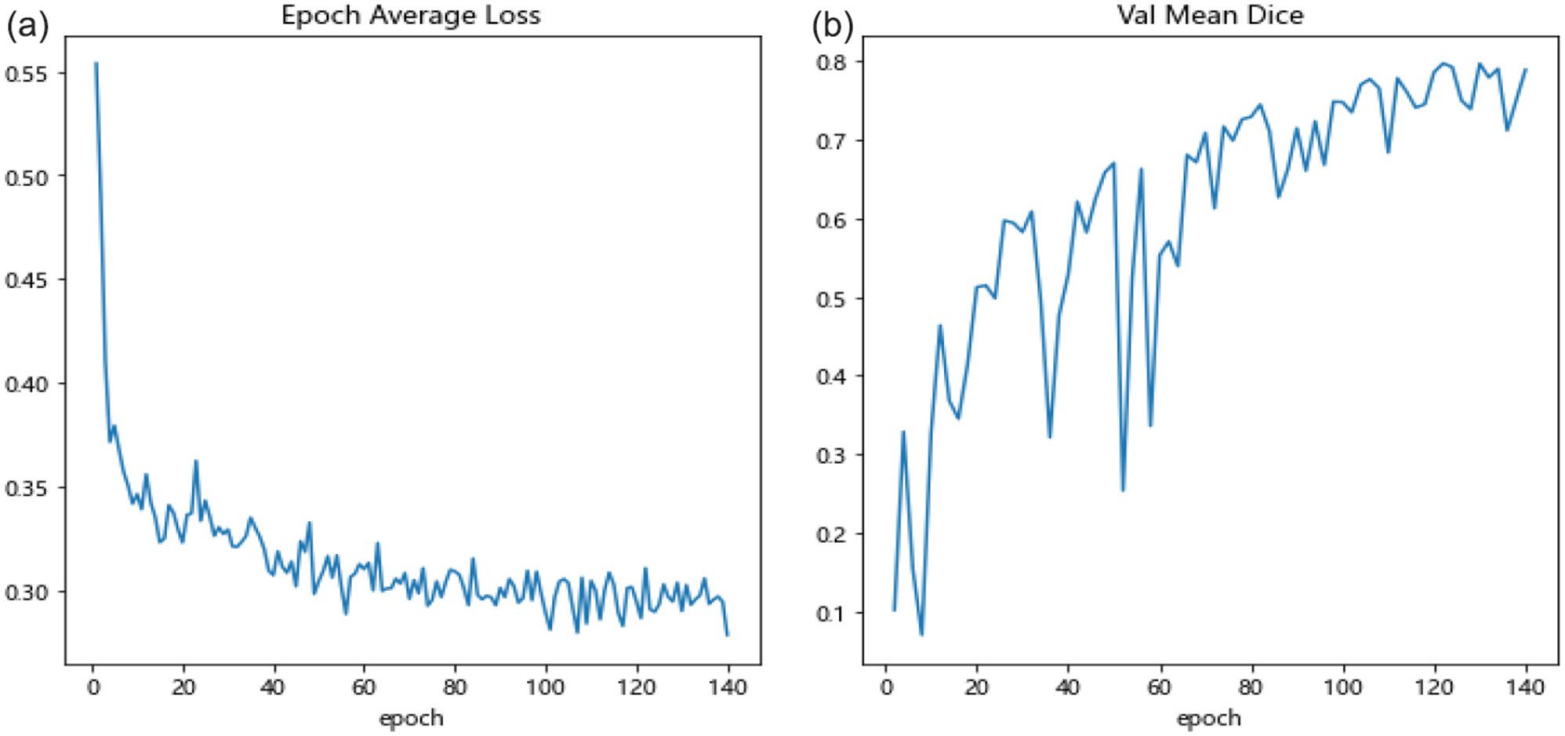
Epoch average loss **(a)** and mean *Dice* score **(b)** of validation

In this paper, an improved hippocampal segmentation model was proposed by introducing a filling technique to 3D-UNet. Figure 5 shows that the trend curves of the average loss and mean *Dice* coefficient of validation vary with the number of epochs. As illustrated in Fig. 5 (a) and (b), a continuous decrease in loss is observed around the 140^th^ epoch. In addition, the loss reaches a minimum at this point. Moreover, the *Dice* coefficient gradually increases and reaches a peak around the same epoch.

To assess the effect of the filling technique, we compared the performance of models established using the original networks VNet, SegResNet, UNetR and 3D-UNet and models constructed by introducing the filling technique into different original networks in this paper. A comparison of the performance of the different models is shown in Table 3.

**Table 3:** Comparison of segmentation performance for different models

Network model	Dice score	mIoU	OSR	USR	ASD	HD
VNet [55]	0.770	0.634	0.097	0.197	1.901	28.963
Filling technique + VNet	0.776	0.642	0.092	0.207	0.640	6.821
SegResNet [56]	0.761	0.618	0.076	0.248	3.112	75.963
Filling technique + SegResNet	0.771	0.630	0.068	0.259	0.600	7.806
UNetR	0.800	0.668	0.104	0.159	1.183	41.672
Filling technique + UNetR	0.802	0.670	0.102	0.160	0.654	5.157
3D-UNet [44]	0.5457	0.3794	0.3618	0.1281	63.3934	169.2131
Filling technique + 3D-UNet	0.7989	0.6669	0.0666	0.2218	0.5733	5.1235

*Note Dice* score is the *Dice* score similarity, *mIoU* is the mean intersection over union, *OSR* is the oversegmentation ratio, *USR* is the undersegmentation ratio, *ASD* is the average surface distance, and *HD* is the Hausdorff distance

As shown in Table 3, the model established by introducing the filling technique into 3D-UNet performs better than the original 3D-UNet base model. The *Dice* coefficient is 0.7989, and the *mIoU* is 0.6669, which are higher than those of the original 3D-UNet-based segmentation model. The *P* value of the *Dice* score was 5.9605 × 10^− 8^ < 0.001 according to the Wilcoxon signed-rank test, indicating a significant improvement after introducing the filling technique into the 3D-UNet-based model. In addition, the *OSR*, *ASD* and *HD* were 0.0666, 0.5733 and 5.1235, respectively, which are lower than those of the original 3D-UNet-based model. Furthermore, for other segmentation networks, such as VNet, SegResNet, and UNetR, we found that the performance of the models improved when the filling technique was added to the base models. Specifically, the *Dice* score and the *mIoU* improved compared to those of the original network-based models. Other metrics, such as *OSR, ASD* and *HD*, also displayed reductions. The findings demonstrate that the proposed method improved the performance of the hippocampus segmentation model and reduced the occurrence of false and missed detections. The model established with the proposed method can improve the precision of hippocampus segmentation based on MR images.

**Fig. 6:**
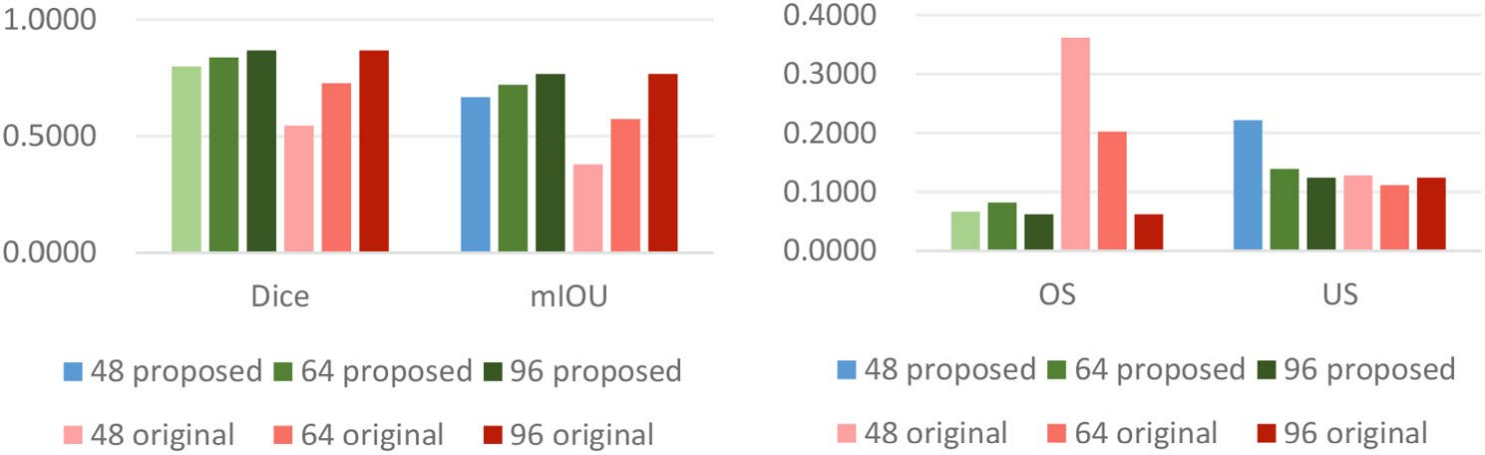
Performance of the 3D-UNet-based model and the proposed model trained with images of different sizes

In addition, we further analyzed the performance of the models trained with images of different sizes. The results are shown in Table 4; Fig. 6.

**Table 4:** Comparison of performance for models established with the 3D-UNet network and the proposed model trained with images of different sizes

size	Model	Dice score	mIOU	OSR	USR	ASD	HD
48	Original	0.5457 ± 0.0828	0.3794 ± 0.0774	0.3618 ± 0.0984	0.1281 ± 0.0580	64.3934 ± 13.7736	169.2131 ± 19.7572
	Proposed	0.7989 ± 0.0398	0.6669 ± 0.0540	0.0666 ± 0.0351	0.2218 ± 0.0849	0.5733 ± 0.1018	5.1235 ± 1.4397
64	Original	0.7271 ± 0.0510	0.5736 ± 0.0615	0.2023 ± 0.0637	0.1116 ± 0.0378	36.9662 ± 7.9704	176.8759 ± 13.2424
	Proposed	0.8371 ± 0.0254	0.7207 ± 0.0370	0.082 ± 0.0372	0.1392 ± 0.0447	0.5319 ± 0.1151	4.3937 ± 1.4050
96	Original	0.8674 ± 0.0257	0.7668 ± 0.0392	0.0621 ± 0.0274	0.1241 ± 0.0445	0.4125 ± 0.0752	3.9032 ± 1.3248
	Proposed	0.8674 ± 0.0257	0.7668 ± 0.0392	0.0621 ± 0.0274	0.1241 ± 0.0446	0.4124 ± 0.0750	3.9665 ± 1.3177

Table 4; Fig. 6 show that as the size of the input image was gradually increased from 48 × 48 × 48 to 64 × 64 × 64 and 96 × 96 × 96, the performance of the segmentation model improved. When the size of training images was set to 48 × 48 × 48, 64 × 64 × 64 and 96 × 96 × 96, the *Dice* scores of the 3D-UNet-based models were 0.5457 ± 0.0828, 0.7271 ± 0.0510 and 0.8674 ± 0.0257, respectively, displaying a gradually increasing trend. In addition, the mIoUs were 0.3794 ± 0.0774, 0.5736 ± 0.0615 and 0.7668 ± 0.0392, respectively. Similarly, the *Dice* score and the mIoU of the proposed model gradually increased as the training image size was increased. Furthermore, when the image size was set to 48 × 48 × 48 or 64 × 64 × 64, the performance of the proposed model was better than that of the original segmentation network-based model.

**Table 5:** Comparison of the performances of models established with the original segmentation network and with the fusion of the filling technique and the original segmentation network for images of size 96 × 96 × 96

Model	Dice score	mIOU	OSR	USR	ASD	HD
3D-UNet	0.8674 ± 0.0257	0.7668 ± 0.0392	0.0621 ± 0.0274	0.1241 ± 0.0445	0.4125 ± 0.0752	3.9032 ± 1.3248
Filling technique + 3D-UNet	0.8674 ± 0.0257	0.7668 ± 0.0392	0.0621 ± 0.0274	0.1241 ± 0.0446	0.4124 ± 0.0750	3.9665 ± 1.3177
VNet96	0.867 ± 0.026	0.765 ± 0.039	0.061 ± 0.026	0.127 ± 0.043	0.408 ± 0.069	3.803 ± 1.068
Filling technique + VNet96	0.867 ± 0.026	0.765 ± 0.039	0.061 ± 0.026	0.127 ± 0.043	0.408 ± 0.069	3.866 ± 1.065
UNetR	0.860 ± 0.023	0.755 ± 0.034	0.076 ± 0.026	0.112 ± 0.038	0.589 ± 0.202	59.437 ± 40.484
Filling technique + UNetR	0.861 ± 0.023	0.756 ± 0.035	0.075 ± 0.026	0.113 ± 0.038	0.459 ± 0.069	3.834 ± 0.709
SegResNet	0.865 ± 0.025	0.763 ± 0.038	0.069 ± 0.030	0.116 ± 0.040	0.427 ± 0.085	7.297 ± 16.789
Filling technique + SegResNet	0.865 ± 0.025	0.763 ± 0.038	0.069 ± 0.030	0.116 ± 0.040	0.423 ± 0.080	3.935 ± 0.870

Table 5 shows that when the size of the input image was increased to 96 × 96 × 96, the performance of the segmentation model improved. For example, the *Dice* coefficient of 3D-UNet-based model trained with images of size 96 × 96 × 96 is 0.864 ± 0.024, which is 7.5% greater than that of model trained with images of size 48 × 48 × 48 for the same network. In addition, the *mIoU* was improved by 10.91%. For other network-based segmentation models, we also found that the model performance improved when the input image size was increased. A comparison of the original network-based models and models that combined filling and the original network-based algorithms indicated that the performance levels were comparable. The improvement in performance achieved with large numbers of training images was not as obvious as the improvement in performance achieved with small numbers of training images. The possible reason is that the large input size mitigates discontinuous segmentation.

**Fig. 7:**
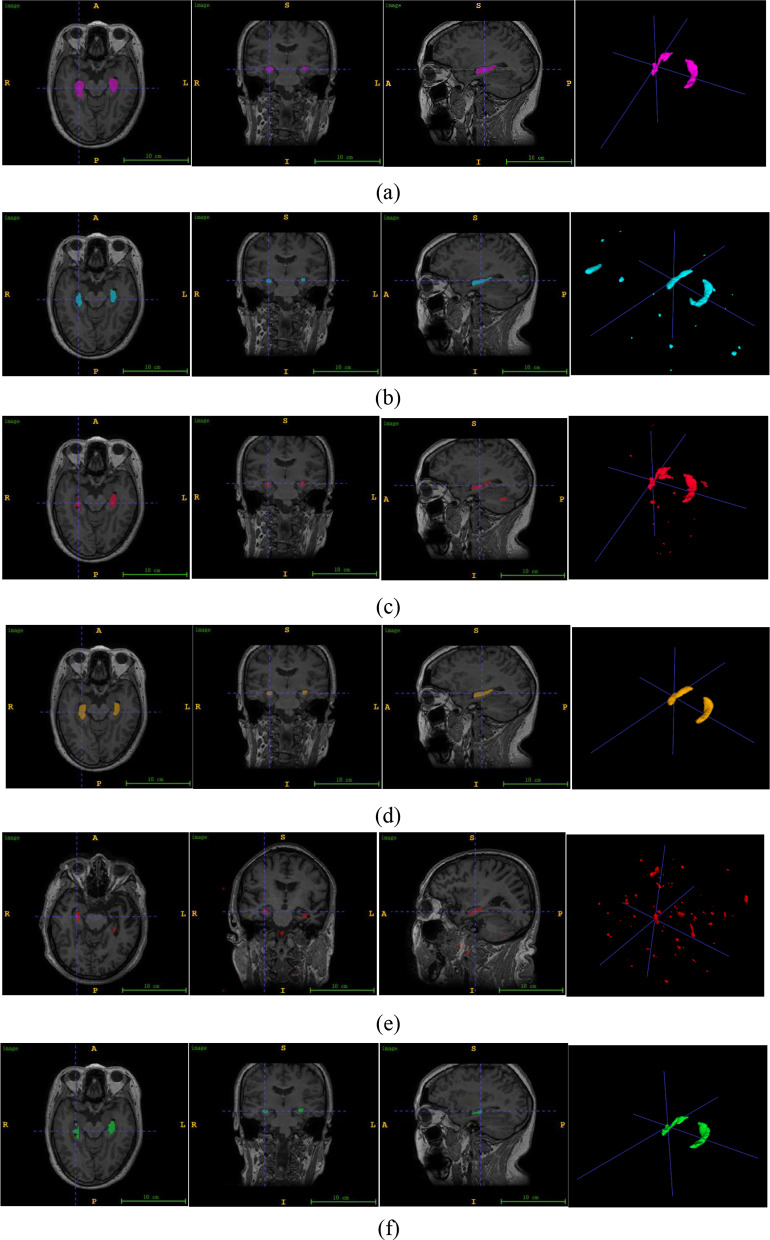
**(a)** is the ground truth. **(b)**, **(c)**, **(d)** and **(e)** show the results of hippocampus segmentation with models established based on VNet, UNetR, SegResNet and 3D-UNet, respectively. **(f)** Results of hippocampus segmentation with the model established by introducing the filling technique into 3D-UNet

Figure 7 (a) shows the ground truth for one MR image. Figure 7 (b) to (f) show the results of hippocampus segmentation with models established based on VNet, UNet, SegResNet, and 3D-UNet and by introducing the filling technique into 3D-UNet with an image size of 48 × 48 × 48.

The performance of different models was assessed, and incorrect segmentation results were obtained with the original network-based segmentation models. By introducing the filling technique into 3D-UNet to establish the segmentation model, the accuracy of segmentation was improved, and details were enhanced and effectively extracted.

## Discussion

Brain metastasis is a common complication of systemic cancer. Brain radiotherapy is the primary method used to prevent and treat intracranial metastatic tumours. However, radiotherapy can cause damage to the hippocampus, leading to cognitive impairment, which severely affects patients’ quality of life. Therefore, accurate segmentation of the hippocampus from MR images is essential for minimizing radiation damage. With the rapid development and application of deep learning in the field of image processing, convolutional neural network-based segmentation algorithms, such as UNet and 3D-UNet, have been developed for hippocampus segmentation based on MR images. One limitation of the 3D-UNet-based model is the discontinuous distribution of the target region in the segmentation results, leading to poor recognition outcomes. Thus, there is a need to improve the accuracy of hippocampus segmentation models. In this study, we propose an improved model by introducing a filling technique to 3D-UNet for hippocampus segmentation based on high-precision MR images. Four deep learning-based models built on the basis of VNet, SegResNet, UNetR and 3D-UNet were constructed to facilitate analysis and comparison with our improved segmentation model. This work has several unique characteristics.

First, a filling technique is introduced into 3D-UNet. The results show that the performance of the model established by introducing the filling technique into 3D-UNet is improved. Notably, there was an increase of 3.22% for the *Dice* score and 4.47% for the *mIoU*. The *OSR* and *ASD* of the improved model were also better than those of the original network-based model.

Second, in the field of hippocampal segmentation, some improved models have been reported. For example, Tang et al. designed a multichannel, landmark large-deformation diffeomorphic mapping method to segment the hippocampus, which yielded a *Dice* score of 0.76 [57]. Hänsch et al. proposed a CNN-based hippocampal segmentation method that achieved a median *Dice* score of 0.76 [58]. Somasundaram and Genish proposed an atlas-based approach to segment the hippocampus from MRI results, and it achieved a *Dice* score of 0.82 [59]. Lin et al. proposed a 3D multiscale multiattention UNet for hippocampal segmentation, and it achieved a Dice coefficient of 0.827 [60]. Although different test datasets were applied in these studies, the *Dice* score in this study was found to be competitive with those of the reported models.

Third, when the filling technique was used for other segmentation networks, such as VNet, SegResNet, and UNetR, the performance of the models was all improved. The results indicate that introducing the filling technique into segmentation networks can improve the performance of hippocampus segmentation models.

Finally, when the size of the input images was set to 96 × 96 × 96, the performance of the segmentation model improved. The *Dice* coefficient of the 3D-UNet-based model trained with images of size 96 × 96 × 96 was 0.864 ± 0.024, which was 7.5% greater than that of the model trained with images of size 48 × 48 × 48. In addition, the *mIoU* improved by 10.91%. This may be attributed to the fact that the large input size helped avoid discontinuous segmentation issues.

## Conclusion

For patients with tumours, brain radiotherapy is often a necessary treatment. If the hippocampus is not adequately protected during brain radiotherapy, the patient’s cognitive functions may be adversely affected. Therefore, when radiotherapy is conducted, it is crucial to precisely delineate the hippocampus to avoid irradiation, which could reduce the impact on the patient’s cognition. In this paper, a hippocampus segmentation algorithm based on a deep learning network and MR images was studied. The current segmentation networks are limited by low accuracy and lengthy processing times. To address these issues, we collected MR images from patients who had undergone brain 3D-T1 magnetic resonance scans. Then, we performed segmentation experiments with the segmentation network. A filling technique was introduced into the original segmentation network to establish a hippocampal segmentation model. MR images were used to validate the accuracy of our automated hippocampal segmentation model. The experimental results demonstrated that our method can effectively segment the hippocampus and improve the *Dice* score, making it highly valuable for hippocampal segmentation tasks. However, due to the limitations of the dataset, further studies based on additional brain MRI data are needed.

## Data Availability

The data presented in this study are available upon request from the corresponding author.
